# How long is enough? Identification of product dry-time as a primary driver of alcohol-based hand rub efficacy

**DOI:** 10.1186/s13756-018-0357-6

**Published:** 2018-05-16

**Authors:** Miranda Suchomel, Rachel A. Leslie, Albert E. Parker, David R. Macinga

**Affiliations:** 1 Institute of Hygiene and Applied Immunology, Medical University Vienna, Vienna, Austria; 2 GOJO Industries, Inc., One GOJO Plaza, Suite 500, Akron, OH 44311 USA; 3 Center for Biofilm Engineering at Montana State University, Bozeman, MT 59717 USA; 4 Department of Mathematical Sciences at Montana State University, Bozeman, MT 59717 USA

**Keywords:** Hand hygiene, Hygienic handrub, EN 1500, ABHR, Application volume, Dose

## Abstract

**Background:**

The World Health Organization has called for the development of improved methodologies to evaluate alcohol-based handrub (ABHR) efficacy, including evaluation at “short application times and volumes that reflect actual use in healthcare facilities”. The objective of this study was to investigate variables influencing ABHR efficacy, under test conditions reflective of clinical use.

**Methods:**

The test product (60% *V*/V 2-propanol) was evaluated according to a modified EN 1500 methodology, where application volumes of 1 mL, 2 mL, and 3 mL were rubbed until dry. Statistical analyses were performed to investigate the relative influences of product volume, hand size, and product dry-time on efficacy, and hand size and hand contamination on product dry-time.

**Results:**

Mean log_10_ reduction factors (SD) were 1.99 (0.66), 2.96 (0.84) and 3.28 (0.96); and mean dry-times (SD) were 24 s (7 s), 50 s (14 s), and 67 s (20 s) at application volumes of 1 mL, 2 mL, and 3 mL, respectively (*p* ≤ 0.030). When data were examined at the individual volunteer level, there was a statistically significant correlation between dry-time and log reduction factor (*p* < 0.0001), independent of application volume. There was also a statistically significant correlation between hand surface area and dry-times (*p* = 0.047), but no correlation between hand surface area and efficacy (*p* = 0.698).

**Conclusions:**

When keeping other variables such as alcohol type and concentration constant, product dry-time appears to be the primary driver of ABHR efficacy suggesting that dosing should be customized to each individual and focus on achieving a product dry-time delivering adequate efficacy.

## Background

Despite the universal acceptance of alcohol-based hand rubs for routine hand antisepsis in healthcare settings, guidance regarding appropriate application volume has been vague and somewhat conflicting. The World Health Organization (WHO) Hand Hygiene Guidelines recommend a “palmful” of alcohol-based hand rub whereas the US Centers for Disease Control and Prevention (CDC) Hand Hygiene guidelines state that the ideal volume of product to apply to the hands is not known [1, 2]. Regarding product rub-in times (dry-times), WHO guidelines state that product should take 20–30 s to rub until dry whereas the CDC guidelines state that if product is dry before 10 to 15 s, then an insufficient amount was used [1, 2]. To make more accurate recommendations, WHO and others have called for the development of improved methodologies to evaluate efficacy of alcohol-based handrubs (ABHR) to obtain results reflective of clinical use [2, 3]. Recommendations include evaluation at “short application times and volumes that reflect actual use in healthcare facilities”.

Several groups have recently investigated the relationships between key ABHR use variables such as product volume, hand size, product dry-times, and log reduction factors using standard EN 1500 methodology [4–6]. These studies suggest that hand size and product dry-time are important variables, but the relative importance of each in determining ABHR efficacy remains unclear. A limitation of these studies is that EN 1500 methodology requires the test product be rubbed for a specific timeframe and then immediately sampled for bacterial recovery [7]. Because the test product often remains wet on the hands when sampled, the methodology does not reflect product use in clinical settings or allow accurate assessment of the influence of product-dry time on efficacy [8–10]. Our group has developed a modified EN 1500 methodology, where the test product is rubbed until dry as prescribed in ASTM E 2755 [10, 11]. Using this method, we have demonstrated the importance of product volume on mean log_10_ reduction factors (RFs). The objectives of this study were to further evaluate the impact of ABHR volume on efficacy, and to investigate the relationships between hand size, dry-time, and efficacy, using a modified EN 1500 methodology where volunteers rubbed test product until hands were dry.

## Methods

The test product (60% *V*/V 2-propanol; Merck, 1.09634, Darmstadt, Germany) was evaluated at application volumes of 1 mL, 2 mL, and 3 mL, respectively, according to a modified EN 1500 methodology on artificially contaminated hands of volunteers.

Experiments were performed at the Institute of Hygiene and Applied Immunology of the Medical University of Vienna, Austria. The laboratory was accredited according to EN ISO/IEC 17025:2005 and recognized by the national accreditation body “Akkreditierung Austria”. All areas of testing were approved and reported to the Federal Ministry of Science, Research and Economy, Austria. This study was performed in compliance with the World Medical Association, Declaration of Helsinki - Ethical Principles for Medical Research Involving Human Subjects. Ethics board approval was not required based on the classification of *Escherichia coli* K12 (NCTC 10538) as a Risk Group 1 non-pathogenic organism by the German Safety Ordinance on Gene Technology. All participants gave informed written consent.

### Modified EN 1500 methodology

For artificial contamination, freshly washed hands were immersed in a suspension of a specified apathogenic strain of *Escherichia coli* K12 (NCTC 10538) up to the mid-metacarpals for 5 s, and allowed to dry for 3 min. Then, to determine pre-decontamination values fingertips from both hands were rubbed for 1 min in a separate petri dish containing tryptic soy broth (Merck, 1.05459, Darmstadt, Germany). Thereafter, the same 15 persons used each volume (1 mL, 2 mL and 3 mL, respectively) of the test alcohol on contaminated hands in a Latin-square crossover design and rubbed in the alcohol until hands were dry. All three volumes (1, 2, and 3 ml) were tested concurrently in a total of three individual runs. Five volunteers used each volume in each run such that after the three runs, each volunteer had used each volume. All EN 1500 tests were carried out with all test volunteers on the same day. Post-decontamination values were determined as described above for the pre-decontamination values. Neutralizing agents were not necessary in these tests because even dilution with the pure broth without supplement was shown in previous validation tests with the test organism *E. coli* K12 to neutralize any antimicrobial effect of the tested alcohol according to the method described in the former “Standard methods for testing chemical disinfection processes” of the German Society for Hygiene and Microbiology (DGHM) (Status 01.09.2001). Finally, all sampling fluids were diluted as necessary and cultivated on the surface of Tryptic soy agar (Merck, 1.05458, Darmstadt, Germany) complemented with 0.05% sodium-desoxycholate (Merck, 1.06504, Darmstadt, Germany) to inhibit the growth of resident microbial skin flora. The plates were then incubated for a total of 48 h and colony forming units were counted and transformed to a decimal logarithm. The log_10_ counts from the left and right hands of each volunteer were averaged separately, for both pre- and post-decontamination values. The arithmetic means of all individual log_10_ reduction factors (RFs) were calculated.

### Dry-time measurements and hand size calculations

The dry-time interval from when a person began to rub to when the person indicated that her or his hands felt dry was recorded during the efficacy evaluation and, additionally, when product was applied onto uncontaminated hands by using a calibrated stop watch. Dry-times for uncontaminated hands were collected on a separate day from the efficacy evaluation to prevent interference from the hand contamination event. Hand surface area (cm^2^) was measured as described previously [12]. Using the definitions of hand size presented in Pires et al., [5] hand size of the study population was evenly distributed with five volunteers having “small” hands (surface area ≤ 375 cm^2^), five having “medium” hands (surface area 376–424 cm^2^), and five having “large” hands (surface area ≥ 425 cm^2^).

### Statistical methods

Log_10_ RFs were assessed via a mixed effects linear model of dry-time and volume, including the interaction, with random effects for run and order of testing (i.e., cross-over period) that accounted for the experimental design (described above), and a random effect for volunteer nested in run that accounted for the repeated measures from each of the 15 volunteers. Likelihood ratio tests and the Akaike Information Criterion were used to assess the effect of volume on log_10_ RFs after accounting for drying time. The effect of hand size on efficacy was assessed by adding to the model above a covariate for hand size. The effect of hand size on dry-times was assessed with a similar model. The mean log_10_ RFs were compared across the 3 different volumes using ANOVA with volume as a fixed effect and volunteer as a random effect followed by Tukey’s multiple comparison procedure. For each volume, dry-times for contaminated and uncontaminated hands were compared using one-tailed paired *t*-tests; pooled across all volumes, dry-times were compared using ANOVA with volume as a fixed effect and volunteer as a random effect. A “conditional R^2^” was reported that includes the effect of all mixed effects in the model [13]. All calculations were performed using R v3.0.2 [14], packages *nlme* [15], *multcomp* [16], and MuMIn [17]. Individual value, residual, and normal probability plots were used to assess model assumptions and check for outliers. All statistically significant results are reported with respect to a significance level of 5%.

## Results

**Fig. 1:**
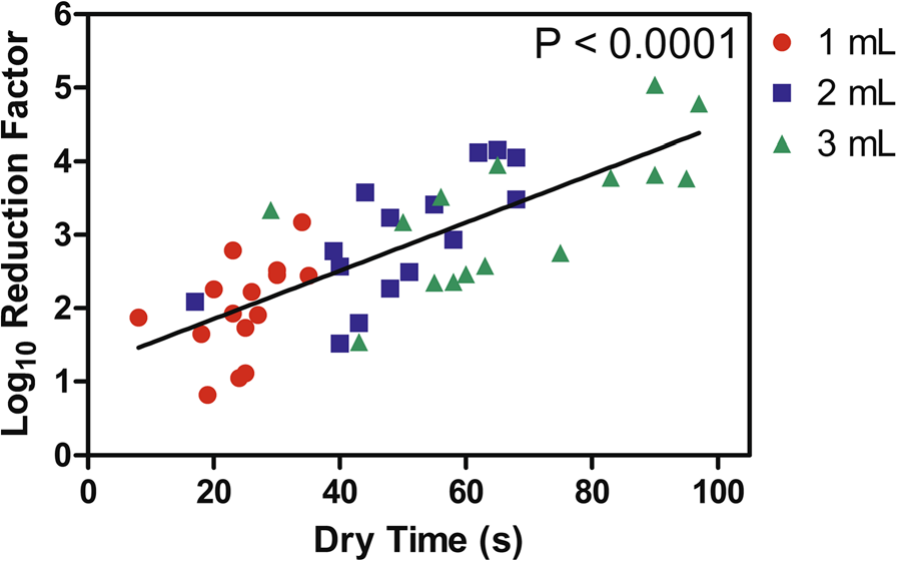
Linear Relationship Between Log_10_ Reduction Factor and Product Dry-Time. The *p*-value indicates a test of correlation

Log_10_ RFs and dry-times of 60% *V*/V 2-propanol were evaluated at multiple application volumes using a modification of EN 1500 where products were rubbed into the hands until dry (Table 1). Mean log_10_ RFs were greater when larger application volumes were used (*p* < 0.0001), however the mean log_10_ RFs between the 2 mL and 3 mL applications were not significantly different (*p* = 0.08). Mean dry-times were also greater when larger application volumes were used (*p* < 0.0001). When individual log_10_ RFs were plotted versus dry-time (Fig. 1), there was a statistically significant linear relationship between dry-time and log reduction factor (*p* < 0.0001), while volume did not have a statistically significant effect in addition to dry-times (*p* = 0.172). Regardless of volume, there was an average increase of 0.29 in the log_10_ RF for every 10 s increase of drying time.

**Table 1:** Influence of Product Application Volume on Antibacterial Efficacy and Dry-Times

Application Volume	Mean Log_10_ Reduction Factor (SD)	Mean Dry-time		*p*-value	
		EN 1500 Testing (SD)	Uncontaminated Hands (SD)	Comparison of Dry-times^a^	Overall Comparison of Dry-times at All Volumes^b^
1 mL	1.99 (0.66)	24 s (7 s)	20 s (5 s)	0.006	0.004
2 mL	2.96 (0.84)	50 s (14 s)	39 s (12 s)	0.005	
3 mL	3.28 (0.96)	67 s (20 s)	59 s (15 s)	0.030	

*N* = 15. All data produced with the same participants

^a^Paired t-test, one-tailed

^b^Repeated measures linear model, one-tailed

**Fig. 2:**
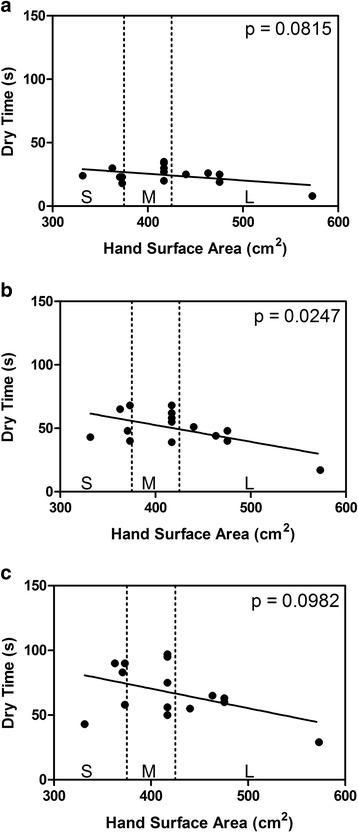
Relationship Between Hand Size and Product Dry-Time. **a**), 1-mL application volume; **b**, 2-mL application volume; **c**, 3-mL application volume. Dashed vertical lines delineate hand size classification (S, small; M, medium; L, large) as presented in Pires et al. [5] The *p*-values indicate a test of correlation at each volume separately

**Fig. 3:**
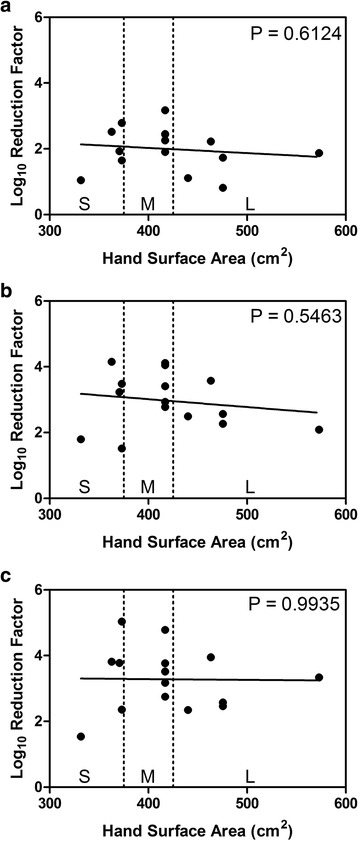
Relationship Between Hand Size and Log_10_ Reduction Factor. **a**), 1-mL application volume; **b**, 2-mL application volume; **c**, 3-mL application volume. Dashed vertical lines delineate hand size classification (S, small; M, medium; L, large) as presented in Pires et al. [5] The *p*-values indicate a test of correlation at each volume separately

The relationship between hand surface area and dry-time is illustrated for each application volume in Fig. 2. There was a weak negative relationship that reached statistical significance only at the 2-mL application volume (Fig. 2b). Overall by itself, hand size did not correlate significantly to dry-time (R^2^ = 8%, *p* = 0.0703). After accounting for the effect of differing volumes, (i.e., after considering the relationship between dry-times and hand size for each volume as in Fig. 2a-c), there was a statistically significant correlation between hand surface area and dry times (*p* = 0.047). However, there was not a statistically significant relationship between hand size and log_10_ RF (*p* = 0.698), even after accounting for dry-times and volumes (R^2^ = 77%, *p* = 0.403) (Fig. 3).

Dry-times for test product applied to hands contaminated according to EN 1500 were statistically significantly longer at each individual volume than those when test product was applied to uncontaminated hands (*p* ≤ 0.030) (Table 1). Overall, it took an average 7.6 s (SD 2.7 s) longer for contaminated hands to dry (*p* = 0.004). At the 3-mL application volume, mean product dry-times were substantially longer than 30 s, regardless of whether product was applied to contaminated hands (67 s) or uncontaminated hands (59 s).

## Discussion

By modifying EN 1500 methodology to better simulate ABHR product application in clinical practice (i.e. rubbing test products until dry) our results provide a better understanding of the relative influence of product volume, hand size, and dry time on ABHR efficacy. While the results in Table 1 confirm previous findings that mean log_10_ RFs increase with product application volume (*p* < 0.0001) [10, 18, 19]; Fig. 1 demonstrates that for the individual volunteer, product dry-time is the primary driver of product efficacy, independent of product application volume (*p* < 0.0001). These results are not surprising since antimicrobial action is a function of active concentration and contact time [20, 21]. As Fig. 1 illustrates, a 2-mL application took longer to dry for some volunteers than a 3-mL application did for other volunteers; and those longer dry-times produced greater log_10_ RFs. From this observation it becomes apparent that the “adequate” or “efficacious” dose is unique for each individual. This concept of individualized dosing is further supported by the large inter-subject variability observed in ABHR efficacy studies [22]. For example, dry-times at the 3-mL application volume, ranged from 29 s to 97 s and log_10_ RFs ranged from 1.54 to 5.04. Since dry-time is the primary driver of efficacy, it is clear that recommending a fixed volume dose will not result in equal (or adequate) efficacy for all individuals. Recommendations for ABHR usage should therefore focus on achieving a specific dry-time (i.e. contact time) as opposed to a prescribed volume.

A negative correlation was observed between hand surface area and dry-time. In other words, the greater the hand surface area, the shorter the dry-time. Even after accounting for all of the observable factors in our study (volumes and hand sizes), the unexplained variability in dry-times was still substantial (100%-R^2^ = 23%) suggesting that other factors beyond hand size influence product dry-time. While more data is needed, we hypothesize that skin moisture content, skin barrier integrity, and amount of hair on the hands may each play a role. Consistent with the findings of two previous studies, we did not find a correlation between hand surface area and log_10_ RF values [5, 6]. These findings do not support the recommendation by Bellisimo-Rodrigues et al. that ABHR use should be customized to healthcare workers’ hand size [4]. These data do suggest that when evaluating ABHR efficacy, inclusion of participants with a broad range of hand sizes may ensure a more representative spread in product dry-times.

Our study has several limitations. First, we used a relatively small sample size which may have limited the ability to detect significant correlations between variables. For example, the relationship between hand surface area and dry-time reached statistical significance for only one of 3 application volumes evaluated and was borderline significant (*p* = 0.047) across the entire data set. And while we did not detect a significant effect of volume on the mean log reduction factor (*p* = 0.172), it is possible that a larger study could detect an effect. Based on the total number of hand rub efficacy measurements (45), the study was conducted over three crossover periods on a single day, which introduced another source of variability. Our statistical model included period and test order as random effects (with subject nested in period as a 3rd random effect). However, the variances associated with period and test order were negligible and therefore not included in our final statistical analysis. Finally, as Table 1 illustrates, the EN 1500 hand contamination procedure, which consists of immersion of hands up to the mid-metacarpals in an *E. coli* broth culture, significantly increased product dry-times compared to dry hands (*p* = 0.004). This phenomenon is likely due to increased moisture and soil introduced to the hands, and has been previously shown to negatively impact ABHR efficacy [19]. To achieve dry-times and efficacy measures more reflective of those in clinical practice, modification of the EN 1500 contamination procedure will be required. The “low-volume” contamination procedure employed in ASTM E2755 has been previously demonstrated to have negligible impact on product dry-times and will be the focus of future experiments [19]. Such an approach has also been proposed by Kampf and may help to better define minimal dry-times needed to achieve an effective hand disinfection [23].

## Conclusions

When considering dosing recommendations for specific ABHR formulations (constant alcohol type and concentration), product dry-time appears to be the primary driver of product efficacy. These data suggest that ABHR dosing should focus on product dry-time (i.e. contact time) to better account for individual variability. The optimal dry-time recommendation remains to be determined and should balance efficacy with healthcare worker acceptance, while create minimal disruption to clinical workflow. Achieving this balance is complicated by the inability of product volumes with short dry-times to meet current efficacy norms. Further studies are needed to determine the clinical significance of these findings (e.g. *minimum* log_10_ RF required to prevent pathogen transmission) and to enable more meaningful ABHR dosing recommendations.

## Abbreviations

ABHR: Alcohol-Based Hand Rub
CDC: Centers for Disease Control and Prevention
RF: Reduction Factor
WHO: World Health Organization
